# A Cross-Layer Secure and Energy-Efficient Framework for the Internet of Things: A Comprehensive Survey

**DOI:** 10.3390/s24227209

**Published:** 2024-11-11

**Authors:** Rashid Mustafa, Nurul I. Sarkar, Mahsa Mohaghegh, Shahbaz Pervez

**Affiliations:** Department of Computer Science and Software Engineering, Auckland University of Technology, Auckland 1010, New Zealand; rashid.mustafa@autuni.ac.nz (R.M.); mahsa.mohaghegh@aut.ac.nz (M.M.); shahbazp@whitecliffe.ac.nz (S.P.)

**Keywords:** cross-layer framework, Internet of Things, secure IoT, general data protection regulation, energy efficient

## Abstract

This survey delves into cross-layer energy-efficient solutions and cutting-edge security measures for Internet of Things (IoT) networks. The conventional security techniques are considered inadequate, leading to the suggestion of AI-powered intrusion detection systems and novel strategies such as blockchain integration. This research aims to promote the development of smart cities by enhancing sustainability, security, and efficiency in the industrial and agricultural sectors through the use of IoT, blockchain, AI, and new communication technologies like 5G. In this paper, we provide a comprehensive review and analysis of secure and energy-efficient cross-layer IoT frameworks based on survey of more than 100 published research articles. We highlight the significance of developing IoT security for robust and sustainable connected systems. We discuss multi-layered security approaches and ways to enhance the energy efficiency of resource-constrained devices in IoT networks. Finally, we identify open research issues and future research directions in the emerging field of cross-layer design for secure and energy-efficient IoT networks. In order to improve cybersecurity and efficiency in smart cities, the research also focuses on developing a secure, energy-efficient IoT framework integrating blockchain, artificial intelligence, and quantum-safe cryptography.

## 1. Introduction

The Internet of Things (IoT) has experienced exponential growth, bringing with it a new era of connectedness and altering our interactions with the surrounding environment. The IoT has an impact on a variety of industries, including healthcare, industrial applications, smart cities, and precision agriculture. But the development of networked devices has also presented several serious difficulties, the most important of which are related to the security and energy efficiency of these systems. We take inspiration for our journey to investigate and resolve these complex issues from an extensive analysis of recent studies conducted in the area. In this paper, we address the following research question: What IoT secure and energy-efficient cross-layer frameworks have been analyzed and surveyed?

The cross-layer framework for secure and energy-efficient IoT integration incorporates many security mechanisms at different IoT architecture layers while optimizing energy usage. It has been analyzed and surveyed. This framework seeks to ensure effective resource use while addressing cybersecurity threats related to Internet of Things devices. It includes energy-efficient routing algorithms and communication protocols in addition to techniques like encryption, authentication, and intrusion detection at various IoT stack tiers. This framework offers a complete approach to IoT security and energy efficiency through thorough analysis and surveying, offering a strong foundation for building and managing IoT devices in a sustainable and secure manner. The availability and privacy of the network layer have become major concerns in recent years, leading to creative methods to prevent attacks and secure critical data [1]. An improved Intrusion Detection System (IDS) has been presented to address contemporary security and privacy challenges by utilizing intelligent architectural frameworks. In the fight for IoT security, these intelligent solutions have emerged as a ray of hope, and our project is motivated by these innovative advancements. At the same time, we turn our attention to the physical layer, also known as the perception layer, where basic security protocols are essential [2]. The physical foundation of the environment is significantly shaped by the Internet of Things. The insights provided by this study regarding the significance of the physical layer are highly relevant to our research aims as we delve deeper into the nuances of IoT security. Additionally, we use a proactive approach, similar to the idea of ongoing active testing and monitoring of the IoT ecosystem, which is supported in [3]. Cities, businesses, and everyday life are all undergoing radical change as a result of the IoT exponential growth. But plenty of difficulties, such as network design complexity, privacy issues, energy efficiency problems, and security vulnerabilities, come along with this expansion of linked devices. This introduction gives a summary of recent research addressing cross-layer energy-efficient frameworks with security measures in IoT.

### 1.1. Motivation of Research

This research is motivated by the pressing need to address important agricultural issues including seed quality and water shortages, which are being made worse by the mounting pressures of population increase and climate change. The rationale behind selecting a cross-layer framework from a wide range of important factors is to improve the energy efficiency of a layered architecture in smart homes, smart agriculture, and medical smart IoT hygiene systems. Firstly, battery life conservation is crucial for medical IoT devices like wearable health monitors, smart air conditioners, smart sprinkler systems, and remote patient monitoring sensors. Battery-powered devices can have their lives extended by using cross-layer frameworks to enhance data processing and communication. In Medical Internet of Things systems, where a dynamic cross-layer structure may prioritize emergency warnings and adjust to changing situations, effective bandwidth management is equally important. In addition, it can facilitate end-to-end encryption, enforce Quality of Service (QoS) regulations, and offer dynamic scalability. The goal of combining IoT, blockchain, and AI [4] is to offer cutting-edge solutions that improve field management and agricultural monitoring, enhancing farming operations’ overall sustainability and efficiency. The study is further motivated by the realization that the swift growth of IoT and Cyber-Physical Systems (CPSs) presents serious security problems [5]. In order to safeguard these dynamic environments, traditional security measures frequently fall short. As a result, sophisticated, AI-driven intrusion detection systems (IDSs) and efficient routing techniques are required, which also improve energy efficiency. Additionally, the project aims to leverage state-of-the-art technologies, like 5G and NOMA-based systems, to revolutionize real-time data communication in sectors like coal mining, providing dependable communication while decreasing energy usage [6]. In addition to addressing current issues in industrial and agricultural applications, this all-encompassing strategy promotes robust, safe, and scalable IoT ecosystems essential for the development of smart cities and vital infrastructure in the future.

### 1.2. Research Challenges

In this survey paper, we address the following research question(s)/challenges. What comprehensive review and analysis can be undertaken to explore cross-layer IoT frameworks for (a) secure and (b) energy-efficient networks?

Justification of research questions: We tested and mitigated Man in the Middle (MitM), eavesdropping, and data manipulation attacks and weaknesses of application layers by comparing real world data acquired by Contiki with the Cooja 2.7 Virtual IoT Simulator. Since technology has advanced, network segmentation and regular firmware updates are necessary in addition to establishing Transport Layer Security (TLS) with the COAP protocol, which is tested for robust security using virtual information. With this method, sensor behavior in controlled environments was simulated using the dedicated Contiki simulator. In particular, we looked at application, network, and sensor layer architecture in order to gain insight into the accuracy and dependability of sensor networks. Our research clarifies the alignment or discrepancies between the two datasets by comparing real sensor data with simulated outcomes. This comparison study provides important insights for improving the security and dependability of sensor networks in a range of architectural contexts by illuminating how effectively these networks function in real-world situations.

We look into the ways sensors are being used in relation to the IoT as a whole and its uses in smart buildings, smart homes, and smart cities. We evaluate and assess the energy efficiency of the Contiki and Cooja 2.7 Virtual IoT Simulator when used with the LEACH, RPL, and ContikiMAC protocols through real-world monitoring and simulated data creation. Because the results provide a thorough understanding of how these simulated networks function in application, network, and sensor layer architecture, they improve environmental surveillance, energy conservation, and overall building efficiency. The implications of this research go beyond improving living and working environments; it may also have an impact on the creation of IoT-driven technologies, offering concrete advantages for enhancing environmental sustainability and constructing energy efficiency in developing IoT networks. Energy consumption is minimized by RPL through the use of objective functions that can prioritize energy-efficient paths and by minimizing the number of transmissions needed for routing. Additionally reducing the control message overhead and supporting other energy-saving techniques, with the help of clustering nodes into groups and rotating cluster heads on a regular basis to balance the energy burden, the hierarchical protocol LEACH seeks to lower energy usage. ContikiMAC is a duty-cycling MAC protocol. Nodes can sleep for extended periods of time and only wake up infrequently to monitor activity because of this feature. Compared to previous objective functions, our approach, which is injected into the Contiki operating system’s core, guarantees a higher level of service quality. The findings show that the new goal function can maintain a packet delivery ratio of more than 97% regardless of density, reduces the network’s average power consumption by about 46%, and reduces the latency and convergence times.

To make a suggested layered architecture more energy-efficient, various cross-layer routing protocols can be created. One strategy is to consider how layers interact and base routing choices on both network and physical layer characteristics. Using routing selection, choice of the best routes that maintain network performance while consuming the least amount of energy possible can be recommended. Utilizing reinforcement learning strategies to improve routing choices is another strategy. We may gain knowledge from the past to improve routing decisions in the future. This method can also consider other performance indicators, such as energy usage. In general, considering how layers interact while optimizing routing choices based on both network and physical layer characteristics is the key to creating an energy-efficient cross-layer routing protocol. By doing this, network performance can be preserved while energy consumption is decreased. A cross-layer framework refers to a system or set of protocols and strategies that operate across multiple layers of the IoT architecture. In traditional layered architectures, each layer operates somewhat independently, but in a cross-layer framework, there is communication and coordination between these layers to achieve specific goals. The primary objective of this framework is to conserve energy within the IoT system. Energy efficiency is crucial because many IoT devices, especially sensors, often operate on battery power. By optimizing energy usage, the devices can function for longer periods without frequent battery replacements or recharging. The IoT systems are typically organized into layers, including the application layer (where data processing and user interaction occur), the network layer (which handles communication and data routing), and the sensor layer (where data are collected from the physical environment). The question focuses on improving energy efficiency across all these layers. So, in essence, the question is enquiring about the strategies, protocols, and mechanisms that can be employed to create a cross-layer framework capable of reducing energy consumption across the entire IoT system, from the application layer’s data processing to the network layer’s data transmission and the energy-hungry sensor layer’s data collection. This framework should aim to optimize energy use without compromising the functionality and effectiveness of the IoT application. Complexity arises when integrating a cross-layer authentication system across various IoT networks and devices. Implementation problems include ensuring smooth compatibility and interoperability while upholding security standards throughout the many tiers of the IoT ecosystem.

### 1.3. Research Scope and Contribution

To address the above proposed research question, we surveyed more than 100 journal and conference papers published over the last five years (2020 to 2024) in well-known databases, including IEEE, ACM, and Science Direct, which appeared in the proceedings of the IEEE Internet of Things Journal, IEEE Transactions on Industrial Informatics, IEEE Sensors Journal, IEEE Transactions on Communications, ACM Computing Surveys, MDPI Electronics, and other relevant outlets. Articles were extracted from the well-known databases as mentioned above. We used search terms including layered architecture, Industrial Internet of Things, IoT networks, IoT cross-layer framework, MAC routing, IoT security, and energy-efficient cross-layer. These search terms are appropriate for our survey paper.

The main contributions of this paper are outlined below. For the secure and energy-efficient cross-layer framework for IoT networks, we carried out a critical analysis and survey of about 109 published research articles.

We categorize the current body of research on the cross-layer framework for IoT that is both secure and energy-efficient according to its independent characteristics. To achieve this, we concentrate on an examination of the routing and multiple access protocols, network resource management, and energy efficiency of Internet of Things networks. A substantial amount of work has gone into the creation and implementation of the next-generation autonomous cross-layer framework that is secure and energy-efficient. We identify areas that need further research, such as the coverage of cross-layer secure and energy-efficient IoTs, cross-layer architecture, quality standards, Industrial IoT, MAC and routing protocols, cross-layer energy-efficient frameworks, and energy-efficient lightweight protocols.

### 1.4. Summary of Existing Surveys

The fields of IoT and cyber-physical systems (CPSs) are explored in the studied literature, emphasizing the shortcomings of conventional security techniques in dealing with the dynamic issues in these areas. The research highlights the necessity for intelligent systems like AI-driven intrusion detection systems (IDSs) for continuous monitoring and adaptability to emerging threats, while traditional security relies on established protocols and perimeter defense. It also explores areas that are frequently missed by conventional approaches, such as multi-layer privacy, cross-layer strategies, and cutting-edge technologies like blockchain and security. Furthermore, the literature presents approaches for risk identification and mitigation, broadening its scope to include cyber-physical systems. Environments related to Industry 4.0 are studied, emphasizing the necessity of thorough risk assessments at several levels. The paper also highlights the significance of strong security solutions for industrial networks and smart cities, including contemporary methods such as encrypted communication, machine learning-based threat detection, and authentication. This paper investigates ways to improve communication performance in the presence of random wardens through the use of Phase Array (PA) and Linear Frequency Diverse Array (LFDA) beamforming in covert millimeter-wave communications. The work demonstrates that various beamforming methods can adapt to maximize covertness and enhance covert throughput by optimizing the transmit power and block length [7]. The study also looks into the issues with energy efficiency in IoT networks and suggests solutions like better node location and routing. The literature emphasizes the need for energy-efficient [8] solutions to ensure the sustainability and dependability of IoT operations in our increasingly digitalized world, as well as the significance of developing IoT security technologies to address evolving threats and challenges efficiently. To defend against cyber-attacks, it also suggests next-generation cyber security designs and emphasizes the significance of Industrial IoT security. The evaluation also touches on the deployment of remote monitoring and control systems in many industries, stressing the need for strong encryption and authentication methods. It also discusses issues surrounding smart city challenges and solutions, stressing the significance of data privacy protection via authentication and encryption techniques. Issues related to energy efficiency in IoT devices and networks are also covered, and strategies for cross-layer optimization and energy-efficient routing are suggested as solutions. The literature analysis (Table 1) offers a thorough overview of IoT security and highlights the necessity of developing security technologies to counteract changing risks and difficulties in the IoT environment.

### 1.5. Structure of This Paper

**Figure 1:**
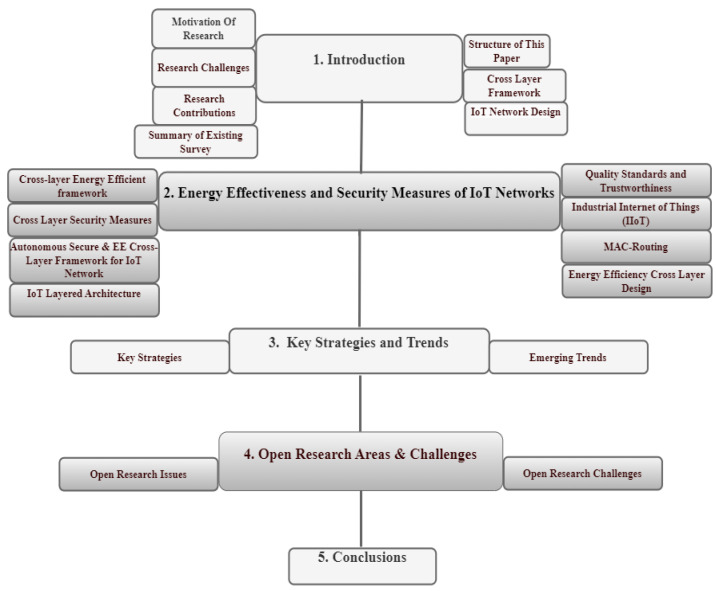
Main structure of survey paper.

The structure and organization of the paper are shown in Figure 1. Section 1 covers the questions, research motivation and challenges of introduction. Additionally, the research contributions related to a cross-layer architecture that is both secure and energy-efficient are analyzed. Additionally, summaries of the existing survey, structure of this paper, cross-layer framework and IoT network design are provided. IoT security measures and energy effectiveness are covered in Section 2. The autonomous, secure, and energy-efficient cross-layer structure is also covered. Moreover, the IoT layered architecture, trustworthy quality standards, Industrial IoT, MAC and routing, and energy-efficient cross-layer design are taken into consideration. Section 3, “Key Strategies and Trends” identifies authentication and encryption, machine learning for threat detection, cross-layer security framework, and energy-efficient routing-optimization. Furthermore, the integration of artificial intelligence, cognitive radio for spectrum efficiency, renewable energy integration, holistic security approaches, and cross-layer optimization are emerging trends. The open research areas and challenges are identified in Section 4, along with a description of the challenges in energy efficiency and the secure cross-layer framework. The paper is concluded in Section 5.

### 1.6. Cross-Layer IoT Framework

The application layer, network layer, and sensor layer are the three basic layers that make up the Internet of Things network architecture. Energy-efficient and security-related components are shown at each layer. Security features like authentication and encryption are part of the application layer, along with energy-efficient application protocols like Message Queuing Telemetry Transport (MQTT) and the Constrained Application Protocol (CoAP) [17].

**Figure 2:**
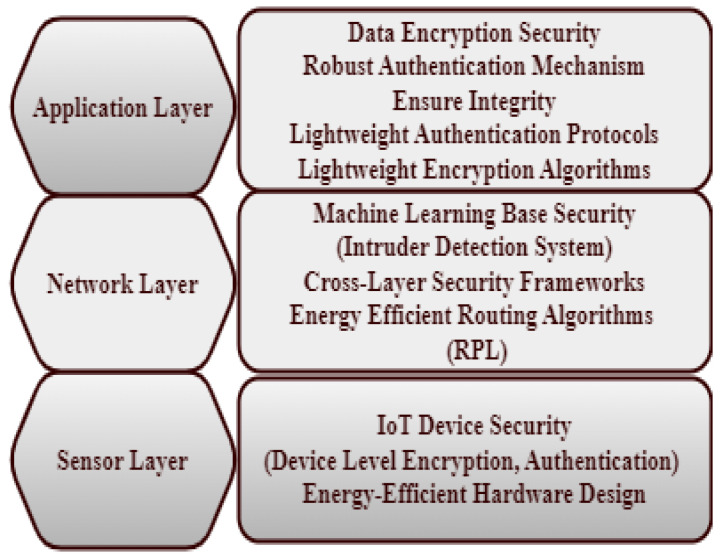
An overview of the IoT cross-layer framework [17].

The network layer incorporates machine learning-based security solutions like intrusion detection systems and middleware security frameworks. This layer also encompasses cross-layer security frameworks and energy-efficient routing algorithms, such as those designed for low-power and lossy networks [17] (see Figure 2). The sensor layer addresses IoT device security with device-level encryption and authentication, along with energy-efficient hardware design considerations. This hierarchical diagram provides a structured overview of how various components contribute to ensuring both security and energy efficiency within IoT networks, as indicated by [17].

### 1.7. IoT Network Design

The performance and scalability of Internet of Things systems are significantly influenced by network design. Cross-layer optimization, in which several network stack layers are coordinated to achieve optimal resource allocation, traffic management, and quality of service, has been emphasized in recent research as being important. Researchers want to address the dynamic nature of IoT environments and accommodate a wide range of applications with different requirements by taking a comprehensive approach to network design [18]. In conclusion, the integration of AI-based analysis, the significance of network design considerations, the search for energy-effective solutions, and the importance of security and privacy safeguards are all highlighted in the recent literature on IoT development. Researchers hope to solve these major issues to fully realize the promise of IoT technology and open the door to a more intelligent, sustainable, and networked future.

## 2. Energy Effectiveness and Security Measures of IoT Networks

### 2.1. Cross-Layer Energy-Efficient Framework

**Figure 3:**
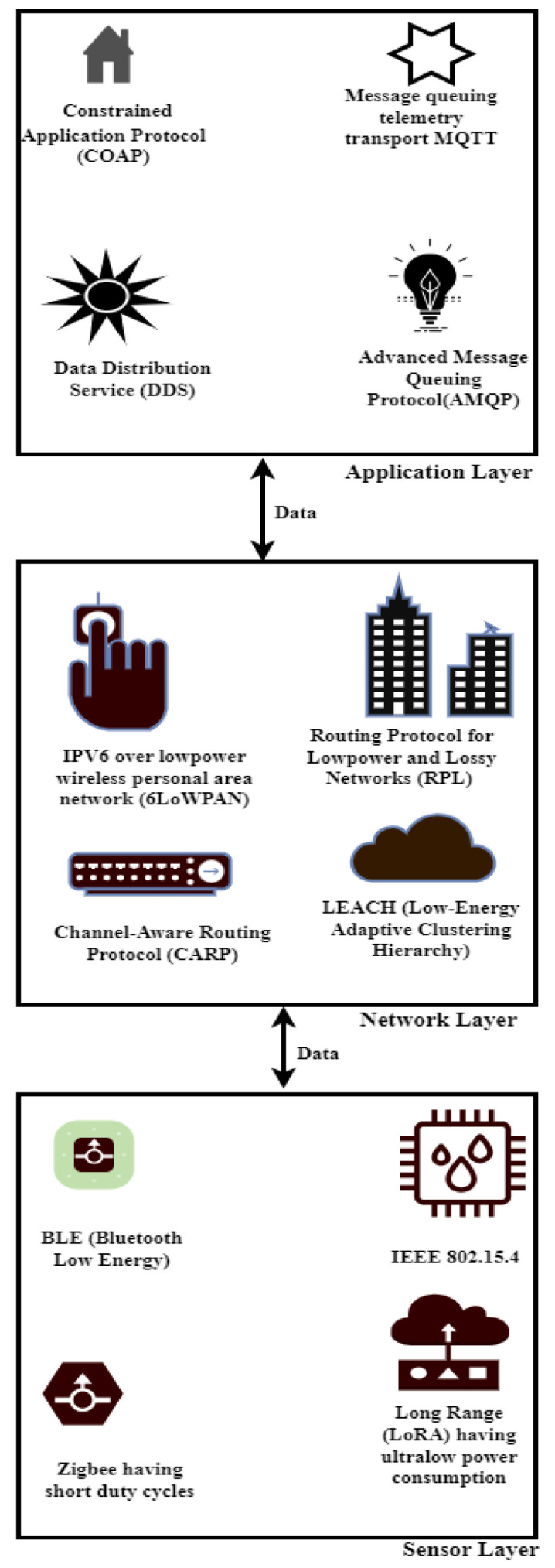
Next generation energy-efficient architecture [17].

Energy efficiency is a crucial consideration for Internet of Things installations, especially because IoT devices are resource-constrained and rely heavily on battery power, necessitating energy efficiency to preserve battery life. According to the literature review, routing algorithms, optimization techniques, and energy-efficient protocols are crucial elements of long-term, sustainable Internet of Things networks. In security implementations, low-power authentication protocols and lightweight cryptographic algorithms are suggested to reduce the computational overhead and energy consumption. In addition, energy-conscious routing plans and optimization techniques maximize the use of network resources, extending the life of Internet of Things devices and lowering their total energy usage (see Figure 3). A collection of low-power, long-range wireless technologies known as LPWAN is intended to support Internet of Things applications that need to be connected over wide geographic areas. The Long Range Wide Area Network, or LoRaWAN, is a popular LPWAN technology that offers low data rates and low-power-consumption long-range communication. It operates in unlicensed frequency bands. Sigfox is another LPWAN technology that offers long-range, low-power communication for Internet of Things devices; Sigfox uses ultra-narrow band modulation and operates in the licensed spectrum. Low-power, wide-area connectivity for IoT devices is made possible by cellular-based LPWAN technologies like LTE-M and NB-IoT (Narrow-band IoT), which take advantage of the current cellular infrastructure.

The main ideas and takeaways from the survey papers are included in this combined introduction of energy-efficient cross-layer IoT networks, which offers a thorough synopsis of the goals and focus of the research (See Table 2).

### 2.2. Cross-Layer Security Measures

Numerous security issues, such as flaws in sensor hardware, cloud computing platforms, and network communication protocols, are highlighted in the literature analysis as being present in IoT ecosystems. Several cybersecurity techniques [25], including intrusion detection systems, authentication procedures, and encryption, are suggested as solutions to these problems.

**Figure 4:**
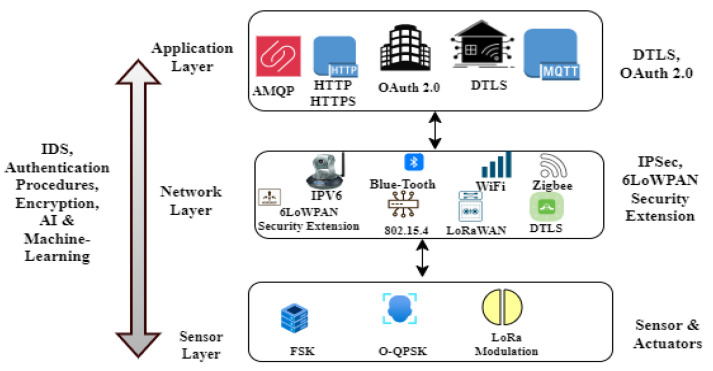
Cross-layer security measures [17].

To effectively counteract complex cyber threats, cross-layer security strategies that integrate network, application, and physical layer defenses are recommended. The resilience of IoT systems against new cyber-attacks is also improved by developments in AI and machine learning, which make it possible to detect and respond to threats with greater sophistication (see Figure 4). For the application layer, it is advised to use the constrained application protocol (COAP), message queuing telemetry transport (MQTT), and Hypertext Transfer Protocol Secure (HTTPS). Recommendations for network layer security include Media Access Control Security (MACsec), IEEE 802.1X, Virtual Private Network (VPN), and Internet Protocol Security (IPsec). Environmental protection, training, physical security policies, secure enclosures, and physical temperature resistance are advised for the sensor layer.

The main idea one can obtain from these published papers is that the secure cross-layer IoT framework provides a thorough synopsis of the goals and focus of the research (Table 3).

### 2.3. Autonomous Secure and Energy-Efficient Cross-Layer Framework for IoT Networks

**Figure 5:**
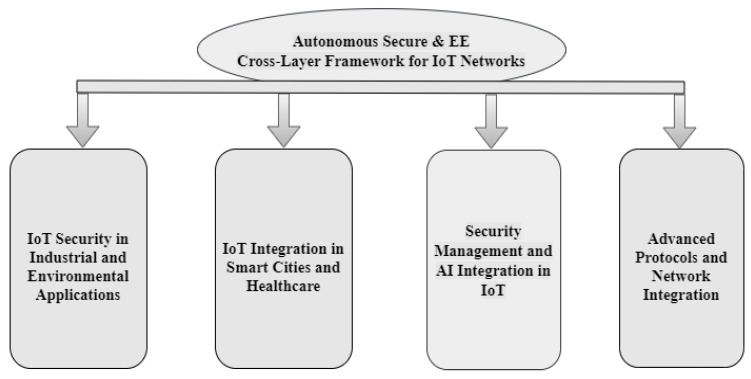
Autonomous secure and EE cross-layer framework for IoT network.

Given the growing sophistication of cyber-attacks, the literature review emphasizes the urgent necessity for strong security measures in IoT systems. To overcome these obstacles, autonomous characteristics must be integrated into a cross-layer framework (see Figure 5). The main independent characteristics that can be included in such a framework are as follows:

(a) Utilizing agentless SIEM modules, such as the Wazuh module, enhances IoT network security by analyzing device traffic and creating alerts for anomalies without requiring endpoint software. This approach successfully protects industrial control systems in Industry 4.0 settings, as demonstrated using the SWaT dataset [30]. The integration of IoT with AI enables continuous data collection and opens new commercial opportunities through intelligent decision-making. Businesses can leverage AI to analyze IoT data with minimal human intervention, enhancing competitiveness [4]. Implementing federated learning models combined with host and network intrusion detection systems within fog computing environments significantly enhances DDoS attack detection and mitigation. This decentralized approach enhances security and lowers the possibility of single points of failure, achieving 89.753% detection accuracy [31]. Using machine learning techniques to identify denial-of-service attacks, such as support vector machines, random forests, and k-nearest neighbours, in IoT networks demonstrates strong detection capabilities, particularly in Information-Centric Networks (ICNs) [32].
(b) Using PCC-RPL and SLF-RPL frameworks improves the security of the RPL protocol in IoT networks by reducing wormhole attacks. SLF-RPL shows better energy efficiency, lower packet loss, and higher attack detection rates compared to PCC-RPL [33]. Integrating Software-Defined Networking (SDN) with Recursive Internetwork Architecture (RINA) enhances IoT network security, flexibility, and scalability. This method facilitates seamless edge-to-cloud connectivity and network function data sharing while maintaining operational integrity [34]. Developing secure, lightweight authentication strategies for low-power IoT devices ensures data privacy and user authentication, which is crucial for applications like Industry 4.0, smart cities, and healthcare [35]. Utilizing learning automata and clustering, this protocol enhances network performance in UV networks by optimizing the cluster node count, service class, and network topology.
(c) Exploring the impact of network softwarization in the industrial sector, this study emphasizes how AI and IoT will play a part in mobile networks in the future, identifying gaps and suggesting areas for further research [36]. The integration of IoT, smart cities, and 5G technology enhances urban living by improving sustainability, efficiency, and responsiveness to citizen demands, transforming urban landscapes [37]. Proposing a Semantic IoT Middleware (SIM) for the healthcare sector [38] addresses data interoperability, heterogeneity, and security using blockchain and AI for optimization and security enhancement. Addressing data security and privacy in E-healthcare applications, ref. [39] integrates blockchain with NuCypher encryption to enhance resource use, resilience, and traceability.
(d) Integrating Raspberry Pi clusters with BME680 sensors Bosch from Ruetlingen, Germany in Kubernetes for environmental monitoring, coupled with OpenID Connect and HashiCorp Vault for dynamic secret management, reduces vulnerabilities and improves responsiveness in IoT installations by 40% and 30%, respectively [40]. The LEMARS model combines heuristic-driven techniques and a Feistel architecture to provide a lightweight encryption solution for secure satellite photography, demonstrating higher attack resilience and quality metrics [41]. Systematizing existing research on enhancing IoT resilience, [42] proposes a taxonomy and classification of resilience mechanisms to address practical concerns in building reliable systems. Examining static, dynamic, symbolic, and hybrid analysis techniques for finding vulnerabilities in embedded firmware, the overview in [43] suggests taxonomies and evaluates these approaches for future research.

### 2.4. IoT Layered Architecture

**Figure 6:**
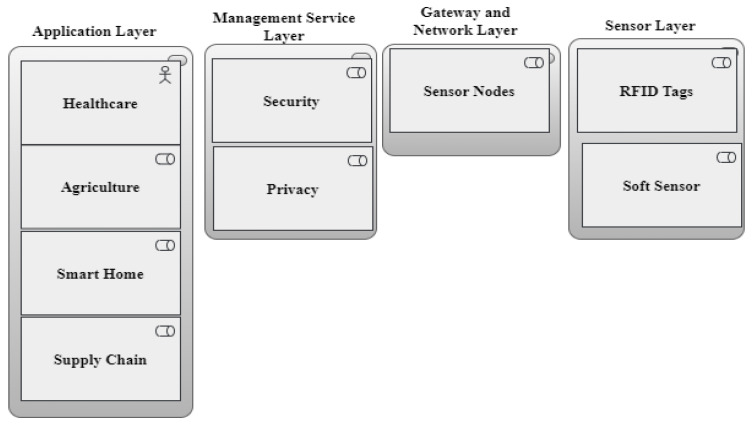
The IoT layered architecture [10].

Traditional security measures are often based on established principles and practices. In contrast, the above-mentioned research explores cutting-edge security measures for the rapidly evolving domains of IoT and CPS. The traditional security typically relies on known protocols and methods, whereas the research advocates for intelligent systems like AI-driven intrusion detection systems (IDSs) to monitor network integrity. While traditional security often focuses on perimeter defense and known vulnerabilities, the research emphasizes continuous monitoring and testing to adapt to emerging threats effectively. Moreover, traditional security may not be well equipped to address the energy efficiency challenges of IoT networks, which the research explores extensively, offering strategies like optimized node placement and (see Figure 6). The research also delves into multi-layer privacy, cross-layer techniques, and innovative technologies like blockchain to enhance security, areas that may not be covered comprehensively by traditional security measures. Furthermore, the research emphasizes the importance of deep learning and application-level security, as well as addressing specific challenges within IoT applications, healthcare, energy management, and efficient routing. It also investigates emerging standards, the role of software-defined security, location privacy in sensor networks, and cutting-edge research on Tactile Internet and 5G networks, which are areas less explored in traditional security approaches. A framework for threat detection of a real industrial network is presented using big data architecture and predictive analytics tools [10]. By obscuring sensitive data and private information, this framework was used to close gaps (see Figure 6), evaluate services and products on an internal level, and share results. In the H2020 ECHO Project, a cutting-edge technological platform was created to exchange and assess cybersecurity data and foster greater trust among various stakeholders.

The review delves into LPWANs like LoRaWAN in IoT, highlighting their appeal for long-range, energy-efficient connectivity. Despite the strengths of LoRaWAN, various challenges persist, including protocol optimization, low data rates, and duty cycle restrictions. To tackle these research challenges, ref. [19] proposes cross-layer optimization, allowing flexibility across protocol layers. It identifies challenges and evaluates cross-layer optimization’s performance in enhancing LoRaWAN for IoT applications.

While privilege escalation and malware infiltrations are security issues, the Internet of Things improves real-time communication. In order to safeguard IoT networks, study [30] suggests an agentless Wazuh security information and event management module (SIEM). The IoT makes it possible to gather data continuously, and when it integrates with AI, it opens up new commercial options for more intelligent decision-making [4]. To be competitive, businesses need to leverage AI developments to analyze IoT data effectively with the least amount of human intervention. Due to its security flaws, the IoT is vulnerable to DDoS attacks, which can seriously harm its finances [31]. To detect and mitigate DDoS attacks in corporate networks, this study uses infrastructure from information-centric networks (ICNs) to address the problem of denial-of-service (DoS) assaults in IoT networks. It suggests using machine learning (ML) to identify denial-of-service (DoS) assaults by contrasting different ML algorithms that perform better, such as support vector machine, random forest, and k-nearest neighbour (KNN) [32]. Using the parental change control routing protocol for low-power and lossy network (PCC-RPL) and the subjective logical framework routing protocol for low-power and lossy network (SLF-RPL) frameworks to reduce wormhole attacks, study [33] improves the security of the RPL protocol in Internet of Things networks. In Contiki OS-based simulations with malicious nodes, subjective logic frame RPL surpasses PCC-RPL in terms of energy efficiency, packet loss, and attack detection. The authors of [34] present an integrated architecture that combines SDN and RINA to improve the security, flexibility, and scalability of IoT.

The study [40] focuses on hardware configuration and strong security benchmarks, integrating Raspberry Pi clusters and BME680 sensors in Kubernetes for sophisticated environmental monitoring. By implementing OpenID Connect and HashiCorp Vault for dynamic secret management and authentication, it achieves 40% fewer vulnerabilities and 30% better responsiveness in IoT installations. Secure device-to-device communication is required due to the growing need for IoT devices in applications such as Industry 4.0, smart cities, and healthcare. Considering the importance of IoT in daily life, data privacy and user authentication must be guaranteed [35]. In this research, a secure, lightweight authentication strategy for low-power IoT devices is proposed, and existing authentication strategies are reviewed. The influence of network softwarization on the industrial sector is the main subject of the survey [36], which examines the function of AI and IoT in future mobile networks. The ways in which these technologies work in concert to improve a city’s sustainability, efficiency, and ability to respond to the demands of its citizens are examined in the book [37]. These technologies are a transformative force that are changing urban landscapes. The study [38] looks at the security void in IoT development and finds that business plans and configurations are not secure enough. In order to improve confidentiality, integrity, and availability while allowing for future developments, it suggests a dynamic security approach using a strong IoT security architecture.

This survey proposed modern solutions like authentication, encrypted communication, and blockchain technology. It introduces novel approaches to threat detection in industrial networks and highlights data security challenges arising from the proliferation of IoT and smart devices. While traditional security approaches are fundamental, the research provides an in-depth, future-oriented exploration of security, and privacy in the dynamic landscape of IoT.

### 2.5. Quality Standards and Trustworthiness

Most of the discussion focused on the growth in IoT devices as well as cyber threats to sensor devices [44]. In addition, the cloud layer, application layer, and physical layer were all investigated. Vulnerabilities and attacks were examined, and cross-layer security was explained. A middleware security approach in the cloud and network, as well as a machine-learning algorithm for attack detection and prevention, are proposed. Cyber-physical systems are made up of intelligent devices that are linked to computer systems and have many cyber vulnerabilities when connected to networks [11]. Many risk identification methodologies are considered, but none consider the interaction of cyber-physical devices. In the industry 4.0 environment, a gap in the literature has been identified. A four-step hybrid methodology is presented. In the first step, ISO 31000, PMBOK, and a risk model are used to identify risk. In the second step, a bottom-up HAZOP strategy is proposed. A physical layer-to-application layer risk analysis is performed. In the third step, the NIST strategy employed a top-down approach. In this step, the physical layer and lower layers of the cyber-physical system were considered. Finally, all risks are combined and analyzed to reduce cyber-physical risk redundancy. The paper [18] emphasizes how IoT can be used for ubiquitous access and real-time analysis, which will enable the integration of IoT systems with social networks to create Social IoT (SIoT). Even though SIoT has advantages, such as improved data trustworthiness and network navigability, there are serious security issues that need to be resolved. Cross-layer security designs, striking a balance between security and energy efficiency, and utilizing graph-powered learning strategies from social networks are some of the suggested answers. This emphasizes how important it is to have strong security protocols and cutting-edge technology in place to protect SIoT ecosystems. The literature study [45] discusses the security issues surrounding the Internet of Things, focusing on how vulnerable networked objects that exchange data over public networks are. It examines new cryptographic protocol standards created to protect Internet of Things communications and assesses how well they work in different application scenarios. It also draws attention to current issues with cryptographic protocols, which is important for improving security in upcoming Internet of Things applications.

**Figure 7:**
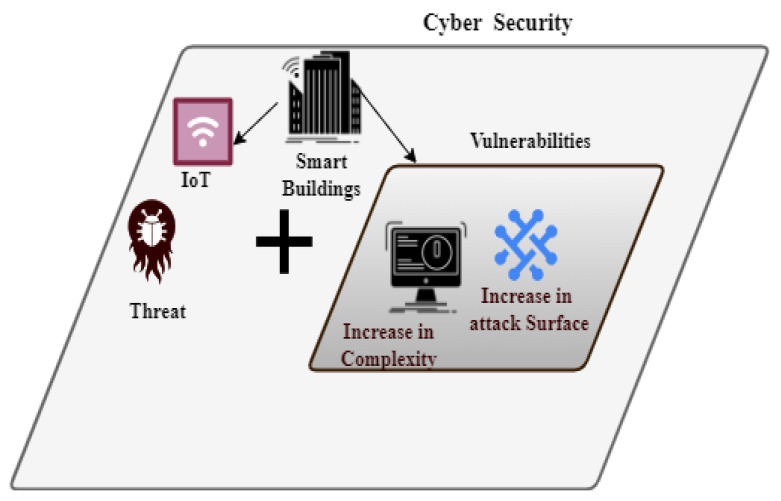
Main parameters of infrastructure risk in smart cities [39].

Experts in cybersecurity must constantly contend with emerging threats that take advantage of flaws in software. To fully realize the potential of IoT, knowledge sharing is essential in building collective resilience against security and privacy challenges associated to IoT [39]. This research highlights the necessity for strong IoT-Cyber security deployment strategies in smart cities by identifying challenges and weaknesses in industries like healthcare and transportation (Figure 7).

By integrating IoT, blockchain, and artificial intelligence (AI), agricultural concerns such as water scarcity and seed quality can be tackled, potentially leading to future success [46]. This study emphasizes seed quality and water management while utilizing blockchain and IoT for effective agricultural field monitoring. The blockchain network improves communication reliability and performance evaluation in prototype design by securing data, promoting community trust, and supporting commercial solutions. In many industries with growing demand, communication technology is essential [41]. The LEMARS model combines heuristic-driven techniques and a Feistel architecture to provide a lightweight encryption and attack-resistant steganography solution for safe satellite photography. Skill optimization is used to cluster nodes, and Deep Q-Learning is used to compute trust [47]. The technique outperforms conventional protocols in simulations in detecting routing assaults such as Black Hole and Gray Hole. Using a blockchain-based control mechanism that combines a software-defined network, ref. [48] tackles congestion in VANETs. In simulations, the suggested approach, which targets connected cars and smart cities, achieves 82% and 98% dependability and efficiency while increasing throughput, packet delivery ratio, energy efficiency, and latency and routing overhead reduction. IoT ecosystems need resilient operability since they are integrated into critical infrastructures and are growing larger and more complex. The paper [42] systematizes existing research on enhancing IoT resilience and analyzes state-of-the-art methods. It also proposes a taxonomy and classification of resilience mechanisms. The Metaverse, made possible by technologies like 5G/6G, XR, and AI, promises immersive experiences but presents serious privacy, security, and trust issues [49]. In order to emphasize the risks connected with AI, this paper examines these problems in AI-XR applications, offers a taxonomy of viable fixes, and provides a case study focused on the Metaverse. Unexplored study areas are noted for future investigation.

The survey [50] looks at edge-based IoT architectures, highlighting the difficulties with decentralized trust management, which is important for safe services, dependable data, and user privacy. It reviews trust criteria, examining blockchain as a trust solution, and examines the performance characteristics of trusted edge IoT systems. The accuracy of trust recommendation models in the Social Internet of Things (SIoT) is evaluated in the survey [51], which also highlights the context-dependent aspects affecting the models’ performance. In order to better understand the research gaps and future prospects for enhancing trust recommendations in SIoT, it suggests a taxonomy that classifies these models according to input attributes and design using the PRISMA approach. Embedded systems are essential in the IoT age, yet they frequently have security flaws because of old or repurposed software [52]. This overview examines the most recent approaches to employing static, dynamic, symbolic, and hybrid analysis techniques to find vulnerabilities in embedded firmware.

With billions of devices connected, the IoT presents significant privacy and security problems, such as handling data and behavioral profiling [43]. This review places these issues in the context of the Internet of Things’ tiered architecture. The authors of [53] present key management, authentication, and encryption techniques that are lightweight and designed for the IoT. They guarantee increased security while using the fewest resources possible to maintain the sustainability of the system. Due to resource limitations and ad hoc topologies, standard cryptography is insufficient for securing the rapidly growing IoT [54]. In response, the cross-layer intrusion detection system IoT-Sentry is presented, which can identify five different types of attacks without requiring additional overhead. To improve attack detection, an innovative cross-layer dataset is also created and ensemble learning is used. IoT-Sentry, using Cooja IoT simulator analysis, achieves an astounding average accuracy of 99% for four out of five attacks, demonstrating a groundbreaking attempt to protect standardized IoT networks from various threats.

### 2.6. Industrial Internet of Things (IIoT)

**Figure 8:**
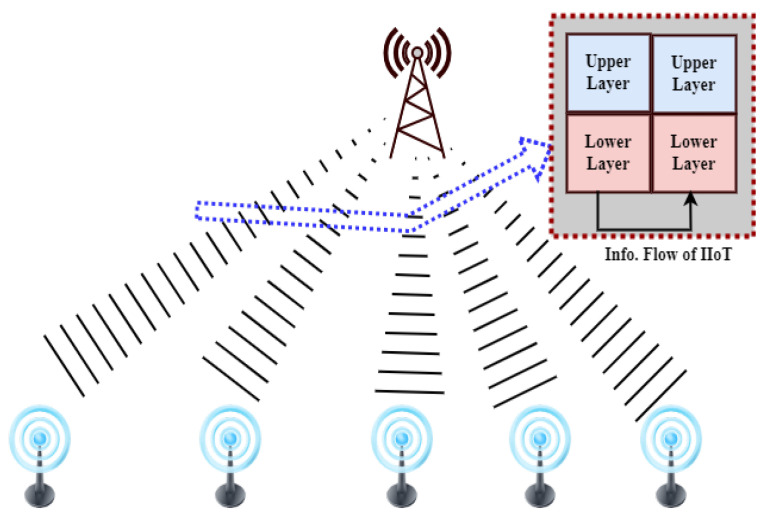
Illustration of 5G-enabled IIoT in Industry 4.0 [60].

For the IIoT to collect monitoring data across industries, wireless sensor networks (WSNs) are essential [55]. Sensitive sensor data security presents difficulties in the resource-constrained IIoT environment, though. In order to enable trustworthy communication amongst linked IoT items, the study [56] surveys IoT authentication strategies that have been presented in the literature. By offering a thorough review and comparison of different methods, together with evaluation models and security analysis, the study seeks to support researchers and encourage more research and development in the area of IoT authentication. The study [57] suggests BF-IoT, a secure communication framework, in response to security issues with Bluetooth Low Energy (BLE)-based IoT networks. In order to prevent spoofing, BF-IoT keeps an eye on device lifecycles and uses special network-flow features for authentication. Continuous device identity authentication is ensured both before and during session establishment by its two-phase defense system. Tests using commercially available IoT devices show that BF-IoT can reliably authenticate devices via sniffing transmission characteristics. The IoT makes it easier to integrate disparate systems, which is essential for smart city applications like traffic and water management [58]. Due to resource limitations, ensuring device authenticity for precise decision making is difficult. The IIoT, crucial for Industry 4.0, grapples with managing real-time manufacturing data amid evolving Internet and telecommunication standards [59]. 5G technology (see Figure 8), while aiding data transmission efficiency, introduces security vulnerabilities in IIoT device authentication. To mitigate this, the article proposes a secure cross-layer authentication framework using quantum walk on circles. This system employs random hash coding on multidomain physical-layer resources for secure device identifier encoding.

With the development of the IoT, which connects everything and everyone, security and privacy have become increasingly important [61]. To reduce security risks in this diverse IoT context, authentication is essential. The review [59] emphasizes the rapid evolution of IIoT in Industry 4.0 and the need for robust security solutions, particularly concerning 5G technology and device authentication vulnerabilities. To address this, a cross-layer authentication framework based on quantum walk on circles is proposed. This framework ensures secure device identification, minimizes decoding errors, and provides high-level security against classical and quantum computers. It achieves ultra-high security and privacy protection with low latency, mitigating potential attacks effectively. The study proposes an innovative cybersecurity approach utilizing a Honeynet architecture to collect real-world network packets and identify attacks. The web-based IDS-AC allows user self-update and performance improvement. Scaling techniques and classifier optimization, particularly Gradient Boosting Classifier, show promise [62]. Future work aims to expand the dataset for comprehensive attack pattern coverage. The literature review [63] systematically examines IoT security research, addressing vulnerabilities, challenges, technologies, and future prospects. Surveying 171 recent publications, it offers a comprehensive overview of IoT development, limitations, and security solutions. The article discusses IoT architecture, common attacks, and mitigation strategies, providing a valuable resource for advancing IoT security technologies and strategies. The authors of [64] suggest a tiered IIoT and industrial control system architecture that optimizes resource allocation and specifies security procedures. The utilization of deception attack simulation and water flow control system modeling for validation highlights the importance of aligning network and security structures for improved security. Interoperable connectivity, which is essential in E-healthcare for real-time patient data management, improves system configuration with the IIoT [65]. Blockchain-based P2P networks and improved Wireless Sensor Network (WSN) lifecycle management maximize resource use [66], resilience, and traceability while tackling scalability and security issues in E-healthcare applications. Decentralized blockchain technology reduces the security threats that smart IoT devices in 5G-enabled networks confront [67]. By using clustered communication and local authentication, a multi-level blockchain security architecture is put forth to improve the security and simplicity of Internet of Things networks. When deployed on Hyperledger Fabric, the model guarantees the legitimacy and effectiveness of the network. IIoT networks with fog computing are used in smart cities to transfer workloads from resource-constrained sensor nodes, which are susceptible to malicious assaults that could impede task completion. Through the identification of malicious nodes and the efficient use of computational resources to ensure timely job completion, the Trust-based Efficient Execution of Offloaded IIoT Trusted tasks (EEOIT) improves fog nodes [68]. Cost, talent, and standardization constraints are the main reasons behind the delayed adoption of IoT in agriculture [69]. The review [70] examines IIoT security and digital forensics, highlighting achievements, challenges, and future directions for cybersecurity. Guidance for researchers and practitioners is outlined to address evolving threats in IIoT ecosystems. Industry 4.0, which emphasizes service-oriented computing for software infrastructure, uses ICT adoption to change production [71]. This paper outlines the function of microservices architecture and points up areas for further research as well as problems. SCADA systems integrate IoT and IIoT for enhanced industrial process control and monitoring [5]. This review explores opportunities and challenges in integrating IIoT with existing SCADA systems. Strong cybersecurity measures are required due to Industry 4.0’s cyber-physical production systems (CPPSs) vulnerabilities in SCADA systems [5]. In order to reduce risks, this study suggests a multilayered structure that includes anomaly detection, encryption, access controls, micro-segmentation, and upgrades for legacy systems. To provide secure remote access for the collaborative demands of IIoT infrastructure, ref. [72] suggests a multi-level authorization architecture that allows for fine-grained access control, scalability, and maintainability. It is implemented using open-source technology and secures the IIoT’s edge and network domains. It has been verified in aircraft situations. The study [60] examines WSNs for Internet of Things security. It examines several attack vectors, approaches for mitigating them, dataset kinds, instruments, and performance metrics across contributions. It highlights the functions of deep learning and machine learning models, providing guidance for future IDS design paths that can effectively secure IoT networks. Computing, energy, and network management are among the issues brought forth by the growing use of IoT network technology in sensitive applications such as healthcare [73]. Based on comparative analysis, it is clear that ESPINA is superior to current protocols, which makes it a prime contender to fulfil 6G wireless communications standards. The authors of [74] investigate how to improve the security of satellite downlink communication in the presence of eavesdroppers in satellite-terrestrial integrated networks by utilizing absorptive reconfigurable intelligent surfaces (RISs). The efficiency of a dual decomposition technique is demonstrated by simulation results, which optimizes beamforming weights and reflective coefficients to maximize secrecy rates. This paper investigates the use of an intelligent reflecting surface (IRS) with simultaneous wireless information and power transfer (SWIPT) to enable resilient beamforming in a secrecy MISO network. Simulation findings verify the efficiency of the proposed strategy [75], which addresses non-convex design difficulties by optimizing secrecy rates among legitimate receivers using convex approximation and alternating optimization techniques.

### 2.7. MAC-Routing

In the context of IoT-driven smart city applications like e-healthcare, ensuring robust security, privacy preservation, and network longevity in Wireless Sensor Networks (WSNs) is crucial, especially during pandemics. To address these challenges, this paper [76] proposes the Cross-Layer and Cryptography-based Secure Routing (CLCSR) protocol. The increasing number of IoT devices in businesses increases security vulnerabilities, which are exacerbated by limitations such as computing capacity and energy resources. The difficulty of protecting IoT from assaults is becoming more and more pressing, especially for mobile devices that need secure data routing methods. To provide optimal routing decisions, the paper [77] presents a secure cross-layer protocol that makes use of MAC layer parameters.

Deploying IoT devices presents issues in the context of Industry 4.0, mostly because low-cost devices are not as capable of providing robust security solutions. In order to solve this, ref. [78] presents a hierarchical authentication and key agreement mechanism that is both lightweight and effective, specifically designed for IoT environments. The authors of [12] propose an energy-aware routing scheme (EARVRT). It utilizes a virtual relay tunnel (VRT), considering route energy, hop counts, and path correlation, However, network longevity slightly decreases compared to the existing method. Future research aims to enhance routing with machine learning and develop intelligent virtual relay tunnels for dynamic adaptation to network conditions. Because of the rapid growth of IoT, modern industries are implementing remote monitoring and control of various IoT devices. In [79], a next generation cyber-ecurity architecture (NCSA) is proposed, as well as industry IoT, for detecting cyber threats [44] and vulnerabilities. A cyber defence authentication mechanism is used to prevent security attacks while a network session is established. A network-wide cryptographically encrypted identity token defence mechanism is established and verified by a virtual gateway system. The proposed NCSA lowers operational management costs while increasing industrial security. While the population of rural areas is declining, the population of cities and the entire world is growing quickly. Lack of resources and limited information sources are both issues that arise [13]. As a result, the need for smart cities increases. Information technology and communication are combined in this idea.

Data security is a major concern because of the exponential growth of IoT and new smart devices as well as the expansion of the Internet [14]. The Internet now has a new meaning thanks to IoT, and managing security is becoming harder. There are two sections to this paper. IoT introduction, building blocks, enabling strategies, and security system requirements are covered in the first section. In the second section, a camera-based case study is examined and its security attributes assessed. The camera system is used for a spoofing attack that can completely control the Smart Camera System (ScS). Following investigation, it is possible to secure systems and address security issues, and to protect customer privacy, beginning with IoT security. For the purposes of using IoT devices, different stakeholders play different roles in preserving information security and privacy goals. The interaction between processors and sensor layers grows as the Healthcare Internet of Things grows [80]. The use of artificial intelligence in healthcare and the Internet of Things is rapidly expanding these days. The Tactile Internet and the Internet of Nano-Things are also recent developments. Emerging technologies as well as how to improve service quality are discussed.

**Figure 9:**
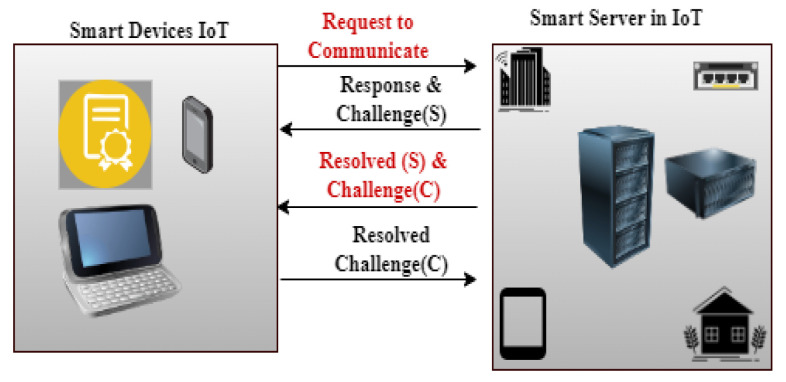
Generalized device-to-server authentication in IoT [83].

Using learning automata (LA) and clustering, this work presents an improved TDMA MAC protocol for UV networks with a focus on cluster node count, service class, and network topology [81]. The suggested protocol exhibits better network performance when compared to conventional TDMA and clustering systems, proving its usefulness for multi-node UV networking. Grid-based routing techniques for wireless sensor networks are covered in detail in this article, with an emphasis on energy efficiency [82]. A comparison analysis and a timeline are presented. With regard to grid topology and energy management in sensor networks, the survey provides insights into design difficulties, challenges, and methodology. The paper [83] introduces a location-aware device-to-server authentication for IoT, enhancing device authenticity using MAC addresses, AES encryption, and MaskIDs generated by a Trusted Authority (TA). It emphasizes the importance of device proximity to servers for authentication, addressing security concerns like man-in-the-middle attacks (see Figure 9). Through analytical and real-world simulations, the proposed method demonstrates superior performance in communication, storage, and processing overheads compared to existing approaches in IoT authentication.

With the support of IEEE 802.15.4-2015 and IEEE 802.15.6-2012 standards [84], low-power biomedical sensors are used in Wireless Body Area Sensor Networks (WBASNs), which are becoming more and more recognized for their potential in patient monitoring [84]. In-depth analysis of current developments in Medium Access Control (MAC) and routing protocols addresses outstanding issues, challenges, and application needs, offering guidance for future work. The results of this research are especially pertinent to the sixth-generation (6G) networks that are predicted to improve quality of service (QoS) and connectivity for a wide range of sensor-based Internet of Things devices. Although the Wireless Medium Access Control (WMAC) protocol guarantees safe data transmission and intrusion detection, clever attackers can circumvent the MAC restrictions that are in place now. In order to secure sensor nodes, the Wireless Interleaved Honeypot-Framing Model (WIHFM) is suggested [85]. This model creates durable security standards and optimum Wireless Sensor Network (WSN) channels. WIHFM introduces honeypot frame traps to improve security by 10% over existing methods like Secure Zebra MAC and Blockchain-Assisted Secure Routing Mechanism (BASR). It does this by isolating distributed attacks creatively and managing the network dynamically. This paper examines distributed MAC protocols in Internet of Things (IoT) and wireless sensor networks (WSNs), with an emphasis on cooperative optimization methods and game theory to improve network efficiency [86]. Key results include significant energy savings and delay reduction through hybrid distributed MAC and cross-layer techniques, as well as an improvement in spatial reuse of 3–29% and an 8% increase in throughput. Methods such as optimum cuckoo search and stochastic methods, along with game theory optimization, demonstrate promising gains in attack detection, resource allocation, and overall network efficiency. The goal of the Automotive Ethernet (AE), the industry’s transition to Ethernet technology, is to increase in-vehicle communication bandwidth for driver-assistance and autonomous systems. But as vehicle connection grows, cybersecurity worries grow, leading to a thorough analysis of AE’s security impact in comparison to protocols like the Controller Area Network [87]. In order to solve industry-specific constraints like low latency, the study concludes that more specialized AE solutions are required, creating a foundation for future improvements. The report outlines important security concerns, mitigation measures, and regulatory mappings. In order to improve security in underwater sensor networks (UWSNs), a secure data aggregation and authentication (SDAA) protocol designed specifically for underwater vehicular wireless networks (UVWSNs) is proposed in [88]. The cluster-based network architecture used by the SDAA protocol improves data communication security and energy efficiency by providing secure cluster head authentication and detecting malicious node attacks. When the SDAA protocol is used in UVWSNs for ship and vehicle monitoring, it shows better network latency and energy efficiency than the current secure MAC techniques. Because of malicious nodes, securing IoT communication networks and node safety is difficult [50]. SDN is used in the suggested Trust Evaluation-based Secure Multi-path Routing (TESM) solution, which includes modules for anomaly handling, multi-path routing with reinforcement learning, and security verification. Based on simulations, TESM is able to localize threats and secure data transmission with just minor increases in latency (12.4%) and throughput loss (5.46%). Although VANETs increase traffic flow and driving safety, their lack of central control makes them susceptible to both internal and external attacks. Black Hole, Gray Hole, and DoS attacks are among the risks that the TDMA-aware Routing Protocol for Multi-hop Communication in Vehicular Networks must contend with [64]. A trust-based architecture is put out as a countermeasure, in which nodes build trust through packet forwarding and channel access behavior. Simulations show how this model greatly lessens the effect of attacks on the network’s functionality.

### 2.8. Energy-Efficient Cross-Layer Design

Coal mines can communicate data in real time by integrating smart sensing devices over the Mine Internet of Things (MIoT) [6]. Reliable communication is ensured by fifth-generation (5G) technology. Energy effectiveness (EE) is increased by optimizing device access and spectrum consumption in a NOMA-based MIoT communication system. Iterative methods tackle issues such as insufficient channel state information, maximizing power allocation, and assigning subchannels while considering cross-layer limitations and quality of service demands. Traditional methods result in high power usage and network breakdowns, motivating the development of energy-efficient routing mechanisms. In the Smart Dust Head (SDH) environment, the latest BS location is disseminated to nodes, enabling optimization through algorithms like Enhanced Oppositional Grey Wolf Optimization (EOGWO) for enhanced network performance [15]. The paper [89] provides a structured overview of the relationship between artificial intelligence and Variable Renewable Energy (VRE) through deep learning (DL) applications. After that, it evaluates DL-based solutions and their suitability while emphasizing important architectures. The study identifies ten DL-based strategies that facilitate VRE integration in power systems. The paper tackles energy-efficient data collection in remote IoT surveillance by designing a Medium Access Control (MAC) layer uplink solution following the Narrowband IoT (NB-IoT) scheduled access scheme. It optimizes energy consumption per uplink frame using Mixed-Integer Non-Linear Programming (MINLP), and introduces a distributed sleep scheduling scheme to enhance delay and energy conservation [20]. Simulation results demonstrate its superiority over existing solutions in terms of delay, buffer length, and energy consumption under high traffic load. The paper addresses energy consumption in IoT devices by proposing ELITE, an energy-efficient cross-layer objective function (OF) integrated into the RPL routing protocol [21]. Unlike existing OFs, ELITE introduces the strobe per packet ratio (SPR) metric at the MAC layer, considering transmission operations’ impact on energy consumption. By selecting paths with fewer strobe transmissions, ELITE reduces average strobes per packet by up to 25% and improves energy consumption by up to 39% The review addresses the energy consumption challenges in wireless networks, particularly focusing on energy-efficient heterogeneous cellular networks (HCNs) [7]. The paper formulates a hybrid joint resource allocation (HJRA) optimization problem and proposes a quantum-inspired political optimizer (QPO) algorithm to address the non-convex nature of the problem, showing superiority in total system EE through synchronous allocation of backhaul bandwidth, sub-channels, and power. The review [90] addresses water scarcity in metropolitan areas and the need for intelligent water distribution systems, emphasizing IoT-based monitoring to manage distribution challenges. In smart city IoT-integrated water distribution systems, it addresses effective delay and energy offloading mechanisms and suggests communication network topologies that are customized to water network design parameters and land cover patterns. Through a case study in Kochi, India, it models delay and energy in IoT-based systems, discusses node categorization algorithms, and identifies optimal fog node placement, achieving up to 40% energy efficiency improvement. The paper [12] addresses various challenges in Flying Ad Hoc Networks (FANETs), emphasizing the importance of routing algorithms due to dynamic topology and energy constraints. It introduces an energy-aware routing scheme based on a virtual relay tunnel (EARVRT) to manage relay nodes, considering metrics like the route energy, hop counts, and path correlation. Evaluation against existing methods demonstrates EARVRT’s superiority in delay, network longevity, energy consumption, and packet delivery rate. The study [91] encompasses the surge in wireless devices, emphasizing their energy consumption concerns and prompting research into energy-efficient solutions. It delves into diverse topics such as computation offloading, CoAP protocols, task scheduling algorithms, and device-to-device communication enhancements, aiming to optimize energy efficiency across various wireless applications. The authors of [16] examine IoT’s connectivity and sustainability challenges due to the surge in connected devices, advocating for cognitive radio (CR) technology as a solution. A cross-layer design is proposed optimizing the modulation order and backoff probability to minimize energy consumption while meeting IoT delay requirements, PR channel availability, and user activities, demonstrating substantial energy reduction and delay satisfaction compared to single-layer approaches. The literature review [29] addresses concerns about energy shortages due to global warming and climate change, focusing on home energy management. The literature review [18] discusses the security concerns in the Social IoT (SIoT), emphasizing the need to balance security with energy efficiency. It suggests employing cross-layer architectures and leveraging graph-powered learning, effective in social networks, to strengthen SIoT security. Additionally, it outlines a study agenda for future research aimed at enhancing SIoT security. Examining the essential elements of low-power, long-range IoT connectivity, this literature study compares LoRaWAN with NB-IoT. NB-IoT is resistant to payload length effects, which is advantageous for buffered applications, according to in-field measurements. On the other hand, LoRaWAN is well suited for longer IoT device lifetimes because of its remarkable energy efficiency, which requires 10 times less energy for similar payload delivery. Enhancing service quality, consistency, and cost management are the main goals of recent developments in IoT-enabled Wireless Sensor Networks (IWSNs) [92]. A hybrid Artificial Neural Network (ANN) Simulated Annealing classifier and optimization based on MapDiminution are used to create an energy-efficient clustering and quick intrusion detection system that addresses security and energy concerns. This method lowers energy usage while achieving a high 97.57% detection accuracy. This work investigates the effective hardware implementation of feedforward artificial neural networks (ANNs) with time-multiplexed MAC blocks by recycling computational resources using approximation adders and multipliers, hence requiring less space and energy [93]. The optimal degree of approximation for hardware correctness is suggested using an algorithm. Comparing experiments with accurate hardware, MNIST and SVHN are real-world datasets for developing machine learning algorithm databases, which demonstrate reductions of up to 50% energy and 10% area with negligible loss in accuracy.

**Figure 10:**
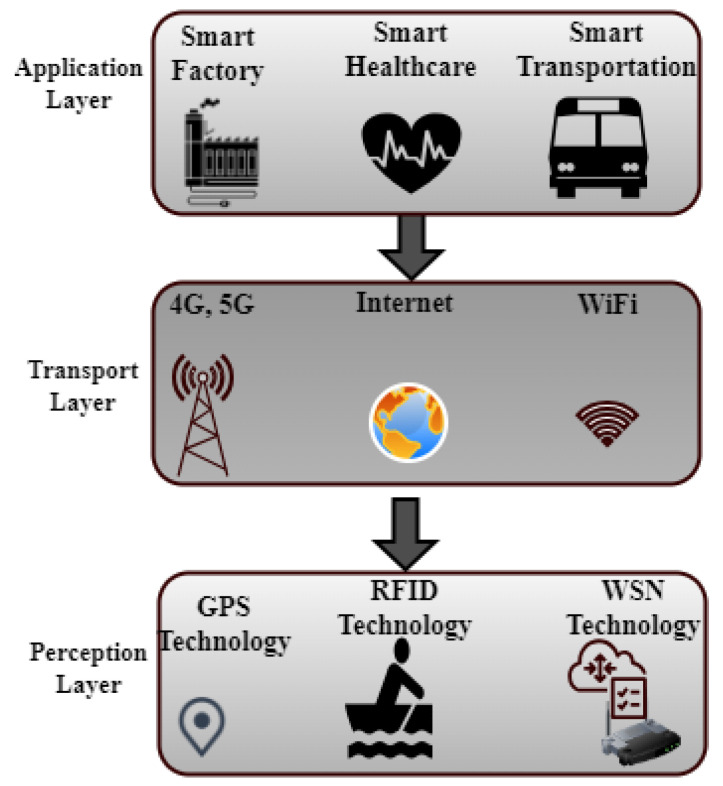
General model of proposed energy-efficient architecture [95].

Energy-efficient wireless sensor networks (WSNs) are crucial for data collecting, as evidenced by the Internet of Things’ exponential expansion [94]. In this study, an energy-efficient cluster-based Lightweight On Demand Ad Hoc Distance Vector Routing Protocol–Next Generation (LOADng) is developed in response to the energy limitations of smart devices. By using LOADng for routing, a seagull optimization method for optimal cluster head selection, and k-means clustering for cluster formation, the technique minimizes power consumption and maximizes network life in comparison to other options. To address the issues with resource allocation that is energy-efficient, ref. [95] emphasizes the significance of IoT in the technological, social, and economic spheres (see Figure 10). A new technique for forest optimization is developed in order to minimize energy consumption and delays in resource distribution, while traditional methods did not account for this factor. MATLAB simulation results show that this method performs better than the generic algorithm (GA), particle swarm optimization (PSO), and distance-based algorithms, with important practical applications and increased efficiency in IoT resource management.

To facilitate coordinated integration in IoT environments, ref. [96] suggests a hierarchical ensemble TinyML scheme that allows individual IoT parts to make decisions that affect the entire system. The two-layered TinyML-based edge computing solution was applied in a smart agriculture use case, showing advantages including decreased energy usage, response times, and wireless transmissions while improving data security and privacy. The assessment demonstrates how well the plan works to produce intelligent, context-aware applications. The swift expansion of IoT in the healthcare sector permits the acquisition of patient data in real-time, while it presents difficulties in managing copious redundant data [97]. An Energy-Efficient Fuzzy Data Aggregation System (EE-FDAS) that reduces energy consumption by reducing typical sensor readings to a single digit is presented in the study as a solution to this problem. Based on NS-2.35 simulations, EE-FDAS outperforms other approaches in terms of aggregation efficiency and energy consumption. The study [98] proposes a clustering technique and Q-learning for work offloading to UAVs, integrating UAVs with IoT to address memory and data processing problems. The suggested approach performs better in terms of data transfer and energy efficiency than the current approaches, especially in emergency situations. The usage of IoT in a smart city for energy-efficient building control is covered in [99], which also suggests a flexible, hierarchical architecture that integrates online services, people, and devices. The suggested model seeks to improve overall smart building operations and to maximize intelligent energy regulation. In the ever-expanding landscape of edge computing, the surge in energy demand poses a critical challenge that demands meticulous management for the advancement of this technology. As edge computing evolves, there is a pressing need to delve into cross-layer architecture, exploring ways to scale energy output while optimizing overall performance [22]. Envisioned as a game-changer in communication, 6G systems are set to revolutionize wireless connectivity for IoT networks [9]. CR technology emerges as a solution for spectrum support, but adapting existing protocols to the energy constraints of 6G IoT devices poses challenges. Energy-efficient protocols, like MQTT, CoAP, Zigbee for sensors and Wi-Fi for networks, are integrated into next-generation IoT architecture [23]. The authors of [8] trace the development of low-power sensors and actuators, promoting RPL-based protocols and lightweight routing strategies; the Internet of Things is reviewed, with a focus on energy efficiency and data dependability. To enhance energy usage, CLEERDTS integrates node location data, optimizes transmit power at the MAC layer, and chooses transmission modes at the physical layer [100]. The security issues of cyber-physical systems, especially smart grids, where Internet of Things devices are susceptible to intrusions, are discussed in [101]. In [102], the power and computational limitations of IoT are addressed by introducing EasyChain, a lightweight blockchain with Proof-of-Authentication (PoAh) for IoE networks. It is implemented in Python and tested on a single-board computer. The authors of [103] introduce the Cluster-based Scheduling and Routing in Geographic Routing Protocol (CSRGR), a dynamic cluster-based duty cycle scheduling for efficient data transmission in resource-constrained WSNs using geographic routing. Study [104] introduces LB-IDS, integrating blockchain for enhanced security in MANETs. The Lightweight Blockchain-assisted Intruder Detection System (LB-IDS) employs lightweight blockchain multifactor authentication for node authentication and multi objective strawberry optimization (MOSO) for optimal route selection, followed by deep-Q learning (DQL)-based IDS for packet classification and blockchain for trust updates [104].

In order to preserve privacy while aggregating data in low-power Internet of Things devices, ref. [105] presents LiPI, a lightweight PPDA method that does away with complicated cryptography and outside dependencies. The focus of this work is on the suitability of lightweight cryptography protocols for Internet of Things security while dealing with devices that have limited resources [106]. It addresses the crucial requirement for effective authentication, privacy, data integrity, and control in Internet of Things networks by comparing the performance of the PRESENT block cipher to alternative lightweight algorithms. Concerns have been raised about the energy efficiency of computing devices and technologies relevant to low-power residential and commercial buildings [24]. A lightweight symmetric cryptographic algorithm is presented in [107]. The reviewed paper proposes an energy-efficient multi-channel MAC framework with a tailored CSMA protocol for CR-enabled 6G-IoT networks. Through joint adaptation of physical and MAC layer parameters, the framework aims to boost IoT network energy efficiency significantly. Numerical results demonstrate up to a 50% improvement compared to a single-channel design, offering a promising solution for 6G-IoT networks’ massive connectivity demands. The surging (WSN-IoT) stands for Wireless Sensor Network on the Internet of Things. which addresses demographic aging and job challenges but faces security threats with its resource-limited devices. Despite its critical role, research on WSN-IoT security is limited [66]. This review zooms in on security challenges, spotlighting a Contiki OS implementation of RPL’s mechanisms. It critically analyzes issues, explores machine learning for management, addresses the architecture, difficulties, network attacks, and goals of the IoT in low-power networks (IoT-LPN). The goal of [108] is to maximize secrecy and energy efficiency in the presence of faulty wiretap channels by examining strong secrecy–energy-efficient hybrid beamforming techniques for satellite–terrestrial integrated networks. For both single and multiple earth station scenarios, two beamforming algorithms with approximate low-complexity solutions are provided to address the nonconvex optimization problem. In order to maximize covert throughput, ref. [7] compares phased array (PA) and linear frequency diverse array (LFDA) beamforming in covert mmWave communication with finite blocklength. The results indicate that improved blocklength and adaptive beamforming improve security against wardens who are spread out geographically.

The related surveys on IoT cybersecurity are summarised in Table 4. The survey scope, IoT security, security-measure implemented, limitations, and the corresponding references are presented in column 1 to 5, respectively.

## 3. Key Strategies and Trends

### 3.1. Key Strategies

**Figure 11:**
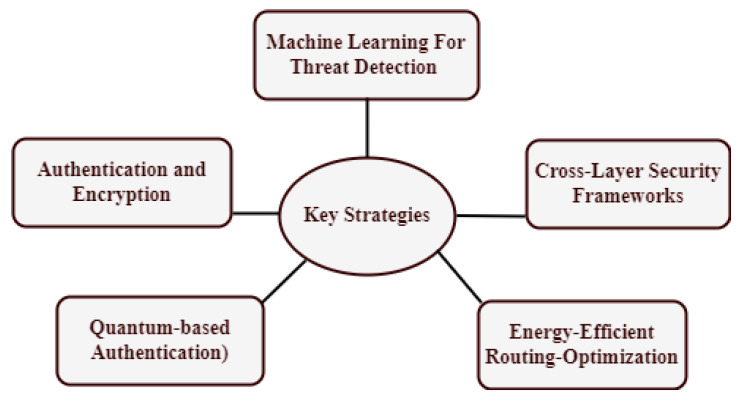
Key strategies of survey paper.

(a) As the number of IoT devices continues to grow rapidly, ensuring robust authentication and encryption mechanisms becomes imperative to protect sensitive data and maintain privacy [14,63]. Authentication mechanisms such as cryptographic protocols and identity management systems help verify the identities of devices and users, while encryption techniques such as symmetric and asymmetric encryption ensure secure communication channels. Additionally, blockchain technology is being explored to provide tamper-proof and decentralized solutions for data integrity and transaction security in IoT environments.
(b) With the increasing sophistication of cyber threats targeting IoT systems, machine learning algorithms are being leveraged for threat detection and prevention [62]. These algorithms analyze vast amounts of data generated by IoT devices to identify patterns indicative of malicious activities or anomalies. By continuously learning from new data, machine learning models can adapt and improve their accuracy in detecting and mitigating security threats, thereby enhancing the resilience of IoT networks.
(c) Cyber-physical systems (CPSs) present unique security challenges due to their interconnected nature and reliance on both physical and digital components [11]. To address these challenges, hybrid security frameworks integrating established risk management methodologies, such as ISO standards with domain-specific risk models, are being developed. These frameworks facilitate comprehensive risk identification and management across multiple layers of CPS architectures, from the physical layer to the application layer. By considering interactions between different layers, organizations can better assess and mitigate security vulnerabilities in their IoT deployments (see Figure 11).
(d) With the advent of IIoT and the proliferation of 5G technology, traditional authentication mechanisms face new challenges related to device authentication vulnerabilities [59]. To address these challenges, cross-layer authentication frameworks based on quantum walk on circles are proposed. These frameworks utilize quantum principles to ensure secure device identification and authentication, thereby mitigating the risks associated with compromised authentication credentials and unauthorized access to IIoT networks.
(e) Energy efficiency is a critical concern in IoT deployments, particularly in resource-constrained environments [12,20,21,22]. Strategies such as energy-efficient routing schemes and cross-layer optimization techniques aim to minimize energy consumption while maximizing network performance and reliability. Additionally, the integration of renewable energy sources with IoT architectures further enhances energy efficiency and sustainability, reducing reliance on traditional power sources and minimizing environmental impact.

### 3.2. Emerging Trends

**Figure 12:**
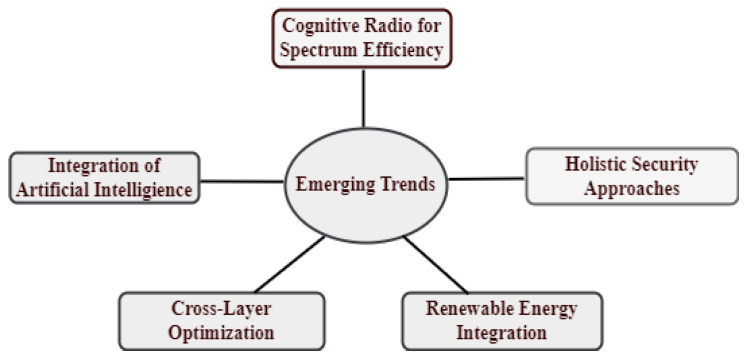
Emerging trends of survey paper.

(a) The integration of artificial intelligence (AI) and machine learning algorithms is emerging as a trend to enhance IoT security and efficiency [62,89]. These technologies enable predictive analytics for threat detection and optimization of energy consumption in IoT systems. By analyzing large datasets generated by IoT devices, AI algorithms can identify patterns and anomalies indicative of security threats, enabling proactive mitigation strategies. Additionally, AI-based solutions facilitate the integration of variable renewable energy sources into power systems, improving forecasting accuracy and grid management.
(b) With the advent of 6G IoT networks, cognitive radio (CR) technology is proposed to optimize spectrum usage and energy efficiency [9]. Multi-channel MAC frameworks tailored for CR-enabled networks aim to improve IoT network performance and connectivity by dynamically allocating spectrum resources based on network conditions and user requirements. These frameworks enhance spectrum efficiency while minimizing interference and energy consumption, thereby enabling reliable and scalable communication in dense IoT deployments (see Figure 12).
(c) The integration of renewable energy sources with IoT architectures is gaining traction for enhancing energy efficiency and sustainability [89]. Deep learning applications facilitate the integration of variable renewable energy sources into power systems, improving forecasting accuracy and grid management. By leveraging AI-based solutions, organizations can optimize energy usage and minimize reliance on traditional power sources, thereby reducing operational costs and environmental impact.
(d) As IoT ecosystems become increasingly complex, holistic security approaches are being advocated to mitigate evolving cyber threats [14,63]. These approaches encompass robust authentication mechanisms, threat detection and encryption protocols. Organizations can increase trust and comply with regulations by implementing comprehensive security measures to protect the confidentiality, integrity, and availability of IoT deployments.
(e) Cross-layer optimization techniques are being explored to improve energy efficiency and performance in IoT networks [22] (see Table 5 for key strategies and trends). By jointly optimizing parameters across different protocol layers, such as the physical and MAC layers, organizations can minimize energy consumption while meeting the diverse requirements of IoT applications. These findings highlight the necessity of comprehensive approaches to cyber-physical systems and IoT security, including improved cybersecurity architectures, all-encompassing security measures, and creative solutions that make use of cutting-edge technology like machine learning and quantum cryptography.

## 4. Open Research Problems and Challenges

### 4.1. Open Research Issues

i It is still very difficult to create strong security measures to keep malware infiltrations, DDoS attacks, and privilege escalation out of IoT networks. For greater application and efficacy, existing solutions, such as the agentless Wazuh SIEM module, offer a good foundation, but they still require improvement [30].
ii It is crucial to create safe routing protocols for Internet of Things networks in order to thwart assaults like denial-of-service and wormhole attacks. Additional optimization is required for attack detection and energy efficiency in frameworks such as the parental change control routing protocol for low-power and lossy networks and the subjective logical framework routing protocol for low-power and lossy networks [33].
iii It is a viable way to improve IoT security and data integrity, particularly in the agricultural and healthcare industries [38].
iv It is crucial to create energy-efficient Internet of Things architectures that can handle a lot of devices without using a lot of power. Measures in this direction include the Semantic IoT Middleware and the hierarchical ensemble TinyML [96].
v It is essential to increase the robustness and resilience of IoT systems to withstand different kinds of cyberattacks and operational failures. More work needs to be done on taxonomies and classifications of resilience mechanisms [72].

### 4.2. Open Research Challenges

i HIDS system and network intruder detection system integration with federated learning to build decentralized, robust security solutions in fog computing environments [31].
ii Ensuring the smooth integration of IoT and AI to manage analytics and real-time data processing. Addressing security and privacy concerns with data while keeping performance high [37].
iii Improving the RPL protocol in order to reduce packet loss, increase attack detection rates, and boost energy efficiency [33]. Customizing secure routing protocols to different Internet of Things contexts ensures scalability and compatibility.
iv Putting into practice blockchain solutions that are lightweight and do not put an undue strain on IoT device resources [102]. Ensuring blockchain’s compatibility with current IoT platforms to enable smooth integration.
v Strong frameworks for data governance that strike a compromise between the requirement for data accessibility and privacy [69].
vi Efficient encryption and secure authentication systems that work with low-power Internet of Things devices [83].

## 5. Conclusions

A cross-layer energy-efficient framework with security measures for IoT is surveyed. We have discussed and categorized a cross-layer framework for the IoT that is both secure and energy-efficient based on key parameters, such as routing and multiple access protocols, energy efficiency, and network resources. We also discussed the development of a next-generation cross-layer framework that is secure and energy-efficient. The findings show the importance of intelligent intrusion detection systems, multi-layered security frameworks, cross-layer techniques, and the adoption of advanced technologies like blockchain, artificial intelligence, and lightweight cryptography to safeguard IoT systems. Energy efficiency emerges as a paramount concern, with strategies ranging from energy-efficient network architectures to battery-saving cryptographic solutions. The safeguarding smart city ecosystems are explored, examining contemporary cybersecurity strategies designed for industrial networks in addition to discussing the need for secure data processing and transfer. In this research we focused on energy-efficient IoT network solutions.

Future multidisciplinary approaches that tackle cybersecurity from both a technological and human perspective should be given priority in research on cyber-physical systems and IoT security. To combat quantum attackers, this entails creating sophisticated threat detection systems that make use of machine learning in addition to quantum-safe cryptography. The key areas of effort should be cross-domain security solutions, physical security improvements, and technologies that protect privacy with a focus on standardization, interoperability, and sustainability. The development of more secure, and trustworthy IoT ecosystems is suggested as future research work.

## Figures and Tables

**Table 1:** Summary of existing survey.

Main Ideas	Survey Scope	Cross-Layer Inspired?	Challenges	Reference
Focused on cross-layer optimization in IoT and multi-channel cross-layer MAC framework.	Low-power, wide-area networking protocol (LoRaWAN) in IoT, cyber security management protocol, 6G communication.	Yes	Protocol optimization, data rate, duty cycle, massive connectivity energy constraint.	[9]
Focused on cyber-physical systems and vulnerability modeling.	Cyber-physical systems, privacy-preserving, cybersecurity information sharing.	No	Data security, vulnerability modeling, sensitive information protection.	[10]
Social IoT Security (SIoT), hybrid risk identification methodology, four-step risk identification process.	SIoT security, risk identification in industry, risk management standards and frameworks.	Yes	Security, energy efficiency and comprehensive risk identification and redundancy of risks.	[11]
Focused on IoT security challenges and solutions, Flying Ad Hoc Networks (FANETs), and energy-aware routing scheme	IoT security challenges, routing algorithms in FANETs, Comparison of routing schemes	No	Security vulnerabilities, cryptographic protocols, dynamic topology and high mobility, efficient path selection	[12]
Focused on smart city security and privacy, solutions using blockchain and encryption, and blockchain for security.	Adaptive cybersecurity, IoT and cloud-based security issues, data privacy and security solutions, smart city data management.	No	Real-world network packet collection, machine learning, resource optimization vs. security, decentralized and distributed structure.	[13]
Focused on IoT security, case study on camera-based IoT, and privacy	IoT security, threat analysis for Smart Camera Systems (SCSs), IoT security and privacy.	No	IoT development, security solutions, complexity of IoT security, vulnerabilities in IoT applications.	[14]
Focused on Non-orthogonal Multiple Access (NOMA)-based IoT communication systems, 5G and energy-efficient optimization	IoT networks and communication, energy efficiency and optimization techniques.	Yes	Energy efficiency, complexity of optimization, and quality of service.	[6]
Energy-efficient routing for smart networks, energy-efficient routing, fuzzy clustering and optimization.	Smart energy-efficient routing, routing architectures, optimization techniques.	No	Energy-efficient routing, network performance, network stability, efficient routing.	[15]
Cognitive energy-efficient IoT, spectrum demand, communication protocols.	Cognitive Radio (CR) technology for IoT, cross-layer optimization, performance evaluation.	Yes	Spectrum optimization, energy efficiency, network adaptation.	[16]

**Table 2:** Summary of literature review on energy-efficient cross-layer IoT.

Key Contributions	Performance Evaluation Methods	Limitations	Reference
Increase the energy efficiency of Heterogeneous Cellular Networks (HCNs) by using NOMA, concentrating on energy efficiency, formulating optimization problems, and introducing the quantum-inspired political optimizer (QPO) method.	Optimization of hybrid resource allocation, simulation outcomes (performance assessment of the QPO algorithm).	Algorithm specificity, non-convex problem complexity, use of simulated comparisons, and possible difficulties in real-world deployment.	[7]
Multi-channel MAC design, per-bit energy efficiency maximization, joint adaptation of physical and MAC layer parameters, and numerical results were developed for CR-enabled 6G-IoT.	MAC design with multiple channels and numerical outcomes.	Simulation dependence, possible difficulties with real-world implementation, and limitations in 6G-IoT design.	[9]
Enhanced LoRaWAN for IoT applications, cross-layer optimization, performance evaluation.	Cross-layer optimization of LoRaWAN and challenges faced.	Lack of validation and system optimization.	[19]
Designed energy-efficient MAC solution for Narrow-band IoT (NB-IoT), optimization framework and cross-layer approach	System optimization, distributed sleep scheduling and system simulation.	Reliance on simulation, resource constraints, traffic model assumptions and scalability.	[20]
Integrated energy-efficient routing protocol for low-power and lossy networks (RPL), new routing metric, cross-layer optimization, and path selection improvement.	Energy-efficient cross-layer integration, RPL protocol and simulation.	Limited system evaluation IoT environments, complexity in implementation and MAC layer dependency.	[21]
Energy-efficient routing in Flying Ad Hoc Networks (FANETs), virtual relay tunnels based on an Energy-Conscious Routing Strategy (ECRS), multiple metrics incorporation, path correlation metric and improve route selection.	Virtual relay tunnel energy-aware routing, comparison with current techniques, comparative analysis, and simulation studies.	Limited practical validation; possible compromises between longevity and efficiency, FANET specificity, path selection complexity, and comparative scope.	[12]
Investigated secure data transmission that uses less energy, edge computing energy management, multi-scale grasshopper optimization, and dynamic honey pot encryption solution.	Cross-layer energy optimization, evaluation of encryption and decryption time analysis, and comparison with current methods.	Absence of empirical support, possible complexity in managing several layers, focus on a particular dataset, and limitations in terms of scalability and real-time.	[22]
IoT integration of energy-efficient protocols, mathematical modeling, a cross-layer energy architecture model, and an emphasis on green and renewable energy.	Energy efficiency through the use of MQTT, CoAP, Zigbee, and Wi-Fi; support for multiple Internet of Things applications, mathematical analysis, and power savings estimation.	Focus on the theoretical framework, scalability and applicability, lack of empirical validation, and limited investigation of practical implementation.	[23]
Examined energy efficiency IoT, determining common design elements, and prospective research pathways.	Analyzing energy management hardware and software, and using previous data to make predictions.	Absence of application-specific variables and system validation.	[24]

**Table 3:** Summary of literature review on security measures in IoT networks.

Key Contributions	Limitations	Security Measures?	Reference
Review of machine learning and deep learning techniques for IoT security, systematic literature review (SLR) of AI approaches for IoT cybersecurity, identifying well-liked methods for high efficiency of detection, like support vector machines and random forests (RF).	Framework for network layer intrusion detection, does not have a cross-layer plan, despite advances in AI, there are still security and privacy issues. Intelligent architectural frameworks are required for improved intrusion detection.	Internet of Things devices are secured using artificial intelligence approaches. Utilizing AI-based frameworks for intelligent intrusion detection systems (IDSs) and employing AI techniques to detect cybersecurity risks.	[1]
At the most basic level of security, the physical layer, wireless network layer, and perception were taken into account, global perspective security framework for the personal IoT (PIoT) was proposed, and security vulnerabilities in the PIoT’s perception layer were identified, security regulations and PIoT defenses were created, and research findings were used in real-world projects.	Securing a big, complex cyber-physical network, suggested security framework’s implementation across all layers may provide certain difficulties, securing a big, and suggested security framework’s implementation across all layers may provide certain difficulties.	In order to address PIoT security issues, the autonomous safety system, security audits, residual information protection, intrusion prevention, and data backup are implemented, security framework spans from the perception layer to the application layer, and includes specific security policies and countermeasures.	[2]
Discussion of the IoT ecosystem current threats and vulnerabilities, secure framework for IoT application testing, and framework evaluates IoT applications from the very start.	Difficulty of securing IoT applications, and possible difficulties in monitoring and security testing.	Address security concerns from the start of IoT application development, monitor and test the framework, and evaluate IoT applications.	[3]
COVID-19 IoT security breaches, an examination of IoT’s effects in many industries, breakdown of the service-oriented architectural paradigm into application, network and perception layers.	Increased security attacks on IoT devices, particularly during the COVID-19 pandemic, and a number of privacy issues in quickly evolving IoT ecosystems.	IoT security and privacy concerns, many communication protocols in each IoT tier based on Service-Oriented Architecture (SOA) layers, and an outline of attacks against certain SOA levels and IoT devices.	[25]
Crossed-layer security and privacy in mind, combining AI and IoT for security blockchain adoption for decentralized coordination, strategies for guaranteeing IoT security.	Security at the application layer has not been investigated. Lack of training data, centralized architecture limits, privacy-security concerns, and resource limitations.	Blockchain technology for secure resource and data sharing, AI-based real-time data analysis, dynamically addressing security threats to WSNs and IoT.	[26]
Presented a number of IoT frameworks, a DDoS attack mitigation model, and a network intrusion detection system integrated with federated learning.	Privacy issues with decentralized Internet of Things systems, possibility of more complicated federated training and detection, potential difficulties with real-time and accurate attack detection.	Utilizing both network-based and host-based intrusion detection systems to fully identify attacks, data analysis and anomaly detection for federated learning, distributed architecture to stop volumetric attack traffic, and fog computing detection.	[27]
Discussion of the four tiers of IoT security gateways, analysis of communication standards for information, communication, and telecommunication, and summary of IoT device testing techniques	There may be difficulties in implementing uniform security measures at various levels, and potential complexity in securing various communication standards.	identifying the four security tiers of IoT systems and using security testing techniques to assess IoT systems, and components.	[28]
Examining IoT risks, security needs, and difficulties that combines Software-defined Networks (SDNs) and IoT architecture is proposed, and SDN-based IoT deployment strategies are discussed.	Implementing SDN-based security solutions in various IoT contexts may provide challenges, as will bringing all IoT stakeholders together on a single platform.	SDN-based IoT security solutions are introduced, Software-defined Security (SDSec) is thoroughly explained, and network-based security solutions for the IoT paradigm are highlighted.	[29]

**Table 4:** Summary of related surveys on IoT cybersecurity.

Survey Scope	IoT Security	Security-Measure Implemented	Limitations	Reference
Identification of threats in industrial networks, investigation of a framework that combines AI technologies, and focus on identifying threats in actual industrial IoT sensor networks.	Maintains security and privacy while addressing the problem of identifying threats in IIoT networks.	Predictive analytics and big data architecture.	Used AI-driven predictive analytics in a real-world industrial network, securely exchanging outcomes as part of the H2020 ECHO project.	[10]
Risk identification in cyber-physical systems (CPS), risk identification analysis in CPS industry, and methods for identifying risks across physical and interconnect layers in CPS are examined.	Concentrate on the cyberattacks and security flaws related to industry CPS’s networked equipment and systems.	Developed a hybrid approach to industry risk identification that incorporates well-known frameworks, such as ISO 31000 [12] and created a four-step procedure to methodically find hazards from various sources.	Cyber-physical device interaction is not taken into account, and current methodologies are incomplete.	[11]
Framework for cross-layer authentication to address security issues in 5G-based IIoT, emphasis on protecting initial access and permitting small data transfers without the need for upper-layer authentication.	Emphasizes the need for secure cross-layer authentication systems to reduce the risks associated in 5G-enabled IIoT vulnerabilities.	Addresses 5G device authentication flaws by putting forth a safe cross-layer framework that creates one-time encryption keys and employs quantum walk-based privacy protection.	Security flaws, complexity, scalability, and system performance.	[59]
Cybersecurity for networked devices that uses virtual environment services is known as an adaptive cybersecurity solution.	Addresses the elevated threats brought by the widespread nature of devices connectivity.	Honeynet architecture, machine learning, real-world network packet collecting, and adaptive cybersecurity (AC) systems.	Scalability, data-dependency, planned-dataset extension, and performance enhancements.	[62]
Thorough examination of IIoT ecosystem with an emphasis on digital forensics and security, outline of developments in digital forensics and IIoT security highlighting major achievements and challenges.	Analysis of the IIoT’s dynamic and structural complexity, as well as the vulnerabilities brought about by its ongoing integration.	Examining state-of-the-art security measures implemented in IIoT ecosystems to safeguard operations, reviewing the IIoT-related digital forensics literature, and concentrating on methods and resources to reduce security breaches.	Real-time threat detection, complexity and integration, changing threat landscape, and the need for more research suggested by NCSA.	[70]
The Next-Generation Cyber-Security Architecture (NCSA) for Industrial IoT and IoT-Security research focuses on the application of IoT technologies in industrial automation.	Implementation of next-generation cyber-security architecture and automated cyberdefense.	Attacks, vulnerabilities, real-time protection, cross-layer security, and identity token mechanism.	Limited discussion on cyber–physical device interaction, a lack of thorough performance review, and possible integration.	[79]

**Table 5:** Summary of key research strategies and trends.

Main Contributions	Trends	Application Area	Reference
Predictive analytics, threat detection, big data architecture, protecting sensitive data, and assessment framework.	Closing security weaknesses and boosting stakeholder trust.	Industrial networks	[10]
Energy efficiency, flexibility across protocol levels, LoRaWAN, and cross-layer optimization.	Improves performance and optimizes the protocol	LPWANs and IoT applications	[19]
Introduces a hybrid approach to thorough risk identification that combines PMBOK, HAZOP, NIST, and ISO 31000 techniques to improve analysis and cut down on redundancy.	Minimizes risk redundancy and provides thorough evaluation.	Cyber-physical systems	[11]
Cross-layer security, data accuracy, social IoT (SIoT), and graph-powered learning techniques.	Optimizes energy efficiency and improves network navigability.	SIoT ecosystems.	[18]
Information-theoretic security, random key encryption, lightweight encryption, and key management.	Effective and secure, appropriate for IoT with low resources.	Cyber-Physical Systems (CPS) and IoT	[53]
Ensemble learning, IoT-Sentry, Cooja IoT simulator analysis, and cross-layer intrusion detection.	Excellent detection precision and low overhead.	Standardized networks for IoT	[54]
IoT authentication techniques, hierarchical classification, centralization, and distribution.	Thorough analysis that promotes more study.	Authentication of IoT	[56]
Performance improvement, smart city applications, and lightweight mutual authentication.	Combines efficiency and security, and it performs better than current protocols.	Water, traffic, and smart city management	[58]
Quantum walk, device identifier encoding, cross-layer authentication, and privacy-preserving protocols.	Low latency, high privacy and security.	5G networks and IIoT	[59]
Web-based IDS-AC, real-world attack detection, machine learning, and Honeynet architecture.	Strong attack alerts, user self-updating	Cybersecurity and industrial networks.	[62]
Evaluation of IoT security research, risks, countermeasures, and prospects	Thorough summary that directs further study.	Development of IoT and security solutions	[63]
IoT network technologies in delay, efficient and secured protocol for emerging IoT network applications protocol (ESPINA), key-renewal approach improves security while lowering computational costs.	Energy efficiency, 6G wireless access, better than existing procedures, in line with 6G wireless communications standards, and secure and energy-efficient protocols.	IoT in healthcare, embedded systems, and applications that are sensitive to security.	[73]
Attack detection, secure clustering, lightweight cryptography, and the cross-layer and cryptography-based secure routing (CLCSR) protocol.	Improves network efficiency and protects privacy.	Smart cities, e-healthcare	[76]
Functions that are physically unclonable, key agreement, hierarchical authentication, and cryptography of elliptic curves.	Secure, effective, and resistant to frequent attacks,	IoT and Industry 4.0 settings.	[78]

## Data Availability

No new data were created or analyzed in this study. Data sharing is not applicable to this article.
